# Leicester Panel Doctor Sued

**Published:** 1915-09-11

**Authors:** 


### IN THE LAW COURTS.

### Leicester Panel Doctor Sued.

A case of considerable interest to panel doctors was recently heard at the Leicester County Court, when Emily Daley claimed 11s. 6d. damages from Dr. Roy for alleged negligence in respect of medical treatment. Plaintiff said that on May 15 she was taken ill, and at 6.30 p.m. her father suggested that her mother should go for Dr. Roy, who was their panel doctor. Mrs. Daley deposed to going to Dr. Roy's surgery, which was only two minutes' walk, but Dr. Roy did not arrive until about 7 p.m., and did not visit her daughter until about 8 p.m.; consequently they had to call in Dr. Phillpot, to whom they had to pay 11s. 6d. Mr. Daley said he complained to the Borough Insurance Committee about the matter, and after an inquiry the committee dismissed the complaint. He did not expect them to do otherwise, because the committee was composed of doctors. As Dr. Roy did not arrive, witness went to the surgery about 7.30 p.m., and asked him to come and see his daughter. Dr. Roy replied curtly, and arrived at the house about 7.50 p.m., and did not remain with witness's daughter more than three minutes. He left the house hurriedly, saying, "Come up for a bottle of medicine." Cross-examined by Mr. Ironside, witness admitted having spoken to Dr. Roy at the surgery in rather a "plain manner."

Dr. G. F. Phillpot deposed to having attended the plaintiff. Witness pointed out that panel doctors were under a contract to be present at the surgery during certain hours, except in case of emergency, and in view of Dr. Roy's surgery hours he did not think that the period which elapsed before Dr. Roy saw the plaintiff was an abnormal one.

Dr. Roy said he was under contract with the Borough Insurance Committee to be at his Belgrave Gate surgery from 6.45 p.m. until 7.30 p.m. Mrs. Daley called at the surgery and said, "My daughter has got a pain in the stomach; will you come at once?" Witness replied, "I will come as soon as I can." About 7.30 p.m. Mr. Daley visited the surgery in "great spirit," and spoke to him in a most insulting tone. Mr. Daley said, "If you don't come I shall send for another doctor and make you pay for it." Witness replied that he could not come immediately because other patients came into the surgery just as Mr. Daley left. He found the patient suffering from acute indigestion, due to eating pork. He did not consider the case serious enough to necessitate another visit unless he was sent for. When he arrived at the house he was shown the stairs in a very impolite manner.

Judge Moore Cann said no doubt the parents were in great anxiety about the plaintiff, but the statement made by Mr. Daley about getting another doctor and making the defendant pay was most improper. He dismissed the action. Mr. Ironside asked for costs, because the case was of interest to the medical profession. Had it not been for the fact that the matter had already been before the Insurance Committee, who dismissed it, he would not have asked for costs. Mr. Bulman, for the plaintiff, maintained that no point of law was involved.

The Judge said the case was of interest to panel doctors, and allowed costs on scale B.

